# Complement activation in anti-glomerular basement membrane disease before and after treatment with imlifidase

**DOI:** 10.1093/ckj/sfaf393

**Published:** 2025-12-16

**Authors:** Linnéa Tyrberg, Fredrik Uhlin, Shanavaz Alam, Elisabeth Sonesson, Thomas Hellmark, Anna M Blom, Mårten Segelmark

**Affiliations:** Department of Clinical Sciences Lund, Lund University, Lund, Sweden; Department of Specialized Medicine, Helsingborg Hospital, Helsingborg, Sweden; Department of Nephrology and Department of Health, Medicine and Caring Sciences, Linköping University, Linköping, Sweden; Department of Health Technologies, Tallinn University of Technology, Tallinn, Estonia; Department of Pathology and Laboratory Medicine, Cumming School of Medicine, University of Calgary, Calgary, Alberta, Canada; Hansa Biopharma, Lund, Sweden; Department of Clinical Sciences Lund, Lund University, Lund, Sweden; Department of Translational Medicine, Lund University, Malmö, Sweden; Department of Clinical Sciences Lund, Lund University, Lund, Sweden; Department of Nephrology, Skåne University Hospital, Lund, Sweden

**Keywords:** anti-GBM disease, autoantibody mediated renal disease, cell-free DNA, complement, double positivity

## Abstract

**Background:**

The involvement of the complement system in anti-glomerular basement membrane (GBM) disease is well known but incompletely characterized. The ability of autoantibodies to trigger the classical pathway is evident, while the lectin and alternative pathways also seem to be of importance. We studied complement activation in patients treated with imlifidase, which leads to rapid IgG depletion, to elucidate the role of complement in anti-GBM disease.

**Methods:**

The GOOD-IDES-01 trial included 15 anti-GBM disease patients treated with one dose of imlifidase in addition to standard therapy with 6 months of follow-up. Plasma samples were analyzed for C3, C4 and complement activation products [C4d, C3bBbP and soluble terminal complement complexes (sTCC)]. Ratios of C4d/C4 and C3bBbP/C3 were calculated to correct for plasmapheresis. Serum samples were analyzed for anti-drug antibodies (ADA) directed against imlifidase.

**Results:**

The C4d/C4 ratio decreased rapidly from its pre-dose level, while sTCC decreased more slowly. sTCC and C3bBbP/C3 were above the reference level throughout the trial. We observed a transient increase in C4d/C4 and C3bBbP/C3, but not sTCC, immediately following treatment with imlifidase, which tended to be more pronounced in patients with more pre-existing ADA.

**Conclusions:**

Classical pathway activation decreased rapidly after autoantibody removal by imlifidase and increased again in most of those that experienced a rebound, but terminal complement activation remained elevated throughout the trial. However, due to the small sample size our results must be interpreted with caution.

## GRAPHICAL ABSTRACT

**GRAPHICAL ABSTRACT:**
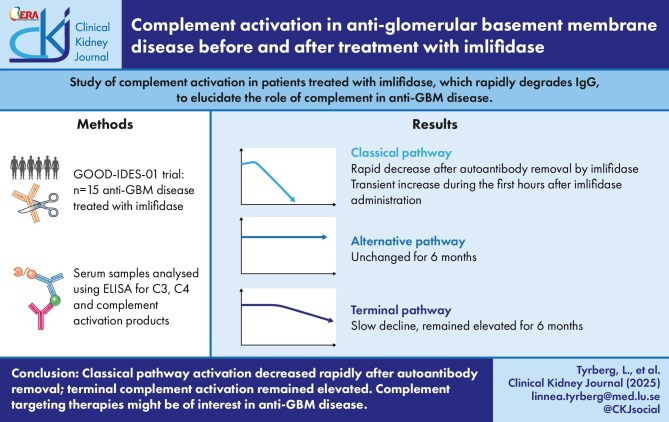

KEY LEARNING POINTS **What was known:**

- The complement system, and the classical pathway in particular, is involved in autoantibody-mediated conditions such as anti- glomerular basement membrane disease, but the involvement of the lectin and alternative pathways, as well as the role of complement activation over the course of the disease, are less well documented.
- The pharmaceutical agent imlifidase degrades all immunoglobulin G *in vivo* within a few hours and was used in the GOOD-IDES-01 trial, offering a unique possibility to clarify the role of the complement system in many autoimmune diseases.
- Therapies based on bacterially derived proteins, such as imlifidase, are immunogenic and high levels of anti-drug antibodies commonly develop after treatment which is expected to have consequences for the potential to repeat such therapies.

**This study adds:**

- Complement activation through the classical/lectin pathways ceased rapidly following autoantibody degradation by imlifidase, but the terminal complement pathway activation remained elevated throughout the trial.
- Increased activity of the classical/lectin pathways in patients with rebound of autoantibodies after imlifidase treatment indicates that there is a relationship between autoantibody level and complement activation.
- There was an early transient increase in classical/lectin pathways activation immediately following imlifidase administration.

**Potential impact:**

- This study strengthens the rationale for removing autoantibodies to reduce tissue damage caused by the complement system in autoantibody-mediated conditions.
- This study suggests that therapies targeting the complement system might be valuable in anti-GBM disease to target the continuing activation of the complement system several months after initial treatment.
- Our findings suggest that repeat doses of imlifidase might be possible within the first few days after the first dose.

## INTRODUCTION

Anti-glomerular basement membrane (GBM) disease, a model disease for autoantibody-mediated conditions, is mediated by autoantibodies towards the α3-chain of the non-collagenous (NC1) domain of collagen IV [1, 2]. The involvement of the complement system in the pathogenesis of autoantibody-mediated conditions has long been recognized [3]. Complement is a protein cascade initiated in response to the binding of antibodies [the classical pathway (CP)], binding of mannose binding lectin (MBL) [lectin pathway (LP)] or spontaneous C3 hydrolysis [alternative pathway (AP)]. Complement acts as a bridge between innate and adaptive immunity through opsonization, recruitment of leukocytes and formation of the membrane attack complex (MAC), also known as the terminal complement complex (TCC) [4, 5]. Anti-GBM autoantibodies are mainly of subclass immunoglobulin G1 (IgG1) and IgG3, and thus capable of activating the CP, but the involvement of the other pathways is less well documented. However, several studies indicate that all three pathways are of importance in anti-GBM disease [6–9]. Furthermore, the pattern and relative importance of complement activation over the course of the disease remains elusive.

The GOOD-IDES-01 trial (ClinicalTrials .gov NCT03157037) evaluated a novel treatment imlifidase in anti-GBM disease with prospective sampling of patients for 6 months [10] and this material presents a unique opportunity to study complement activation in anti-GBM disease. Imlifidase is based on the bacterial enzyme IdeS, which rapidly and selectively cleaves human IgG of all subclasses [11, 12]. The results from the trial were encouraging [10], and currently, the phase III trial GOOD-IDES-02 (ClinicalTrials.gov NCT05679401) and an investigator-initiated phase II trial of imlifidase in anti-neutrophil cytoplasmic antibody (ANCA)-associated vasculitis (AAV) (EudraCT 2021–004706-22) are ongoing. However, as imlifidase is based on a substance from the common bacteria *Streptococcus pyogenes*, most adults have pre-existing anti-drug antibodies (ADA), resulting in a treatment-boosted production of ADA. However, repeat doses might be helpful in the short term in patients experiencing a rebound of autoantibodies shortly after antibody removal by imlifidase or in the long term for example in patients with relapse of AAV.

Cell-free DNA (cfDNA) can be released into the circulation because of cell death, generation of exosomes and microparticles, or the formation of neutrophil extracellular traps (NETs). In inflammatory conditions, there is an increased generation and sometimes impaired clearance, and cfDNA has been suggested as biomarkers in autoimmune disease [13]. NETs are also known complement activators [14] and exposed DNA regulates complement by binding the complement inhibitor factor H [15]. While increased levels of cfDNA are well known in several autoimmune disorders, including systemic lupus erythematosus and AAV [13, 16], their importance in anti-GBM disease is unknown.

This study aimed to investigate complement activation in anti-GBM disease and the relationship between complement activation markers and autoantibody levels as well as double-positivity for ANCA to increase our knowledge of the pathogenesis of autoantibody-mediated conditions. Furthermore, we also aimed to investigate complement activation in relation to treatment with imlifidase. Finally, we wanted to investigate the presence of cfDNA and its relation to complement activation and outcome.

## MATERIALS AND METHODS

### Patients and sampling

Imlifidase treatment in anti-GBM disease was tested in the single-arm phase II trial GOOD-IDES-01 (ClinicalTrials.gov NCT03157037). In this trial, 15 anti-GBM patients from five European countries with an estimated glomerular filtration rate <15 mL/min/1.73 m^2^ were treated with standard therapy for anti-GBM disease and a single dose of imlifidase (0.25 mg/kg) and were monitored for 6 months [10]. Blood samples were collected before imlifidase treatment and prospectively during the trial. The samples were frozen at –80°C at the collection site before transportation on dry ice to the central laboratory, where samples were aliquoted and stored at –80°C until further analysis.

All patients except one received therapeutic plasma exchange (PLEX) before inclusion in the trial. PLEX was restarted due to the return of anti-GBM antibodies after treatment with imlifidase, which is the definition of a rebound, in 10 patients (67%) 1–4 weeks after treatment. One patient received one session of PLEX without any increase in anti-GBM antibody level and was hence not categorized as a rebound.

This study was conducted in accordance with the declaration of Helsinki and was approved by the Regional Ethical Approval Board in Linköping (dnr 2018/518–32) and independent ethics committees and regulatory authorities in all participating countries. All patients provided written informed consent, including exploratory analyses of central laboratory material.

### Measurement of antibodies and imlifidase concentration

Serum samples were analyzed for anti-GBM, ANCA, ANA and ADA antibody levels as well as imlifidase concentration as previously described [10, 17–19] and further detailed in the Supplementary data.

### Measurement of complement components

Plasma EDTA samples were analyzed for C3 levels at the Department of Clinical Immunology and Transfusion Medicine, Region Skåne, Lund, Sweden using nephelometry. Normal range for C3 with this method is 0.76–1.77 g/L. C4 levels were determined using a commercial enzyme-linked immunosorbent assay (ELISA) kit (ab108824, Abcam, Cambridge, UK) according to the manufacturer’s instructions. Normal range (0.13–0.39 g/L) was determined by measurement of 100 healthy donors.

Products of complement activation were measured in plasma using previously described sandwich ELISAs [20, 21] with antibodies reactive to a neoepitope in C4d (CP and LP) and complexes C3bBbP (AP), and soluble TCC (sTCC) (terminal pathway) generated upon complement activation. Reference intervals based on measurement of healthy controls were C4d (*n* = 77) <1.1 mg/L [22], C3bBbP (*n* = 31) <23.4 CAU/mL [21] and sTCC (*n* = 47) <0.9 CAU/mL [23]. C5a was measured in plasma samples using an ELISA kit from Hycult Biotech (Uden, The Netherlands) executed according to the manufacturer’s instructions.

### Measurement of cell-free DNA

DNA was purified from plasma EDTA samples using QIAamp DNA blood kits (Qiagen, Hilden, Germany) according to the manufacturer’s instructions. The amount of cell-free mitochondrial and nuclear DNA (mtDNA and nDNA) was measured using droplet digital polymerase chain reaction (ddPCR), further detailed in the Supplementary data.

### Statistical analysis

For all ELISAs and ddPCRs, double samples were analyzed, and their mean values were calculated in Microsoft Excel (Microsoft 365MSO, Microsoft Corporation, Redmond, WA, USA).

Statistical analysis was performed using non-parametric tests due to the small sample size and all results are presented as median and range. The statistical analysis is further detailed in the Supplementary data.

## RESULTS

### Complement levels in relation to disease activity

#### C3 and C4

Throughout the trial, C4 levels in plasma were within the normal range (0.13–0.39 g/L) for nearly all patients (Supplementary data, Fig. S1A). Likewise, the C3 levels in plasma were within the normal range (0.76–1.77 g/L) for most patients during the trial, though in the lower normal range during the first month (Supplementary data, Fig. S1B). However, some patients displayed values below the normal range before and 6 h after imlifidase treatment and Days 3–29 after treatment, when rebound of anti-GBM occurred. Interestingly, it was almost exclusively patients with rebound of anti-GBM who displayed low C3 values on Day 3–29 (Supplementary data, Fig. S1C).

#### C4d

**Figure 1:**
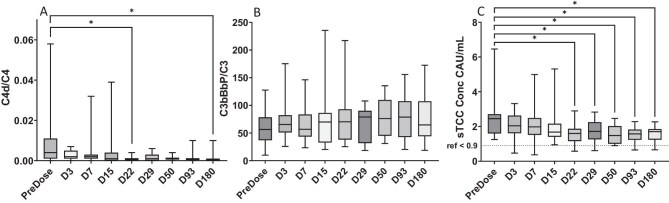
Complement activation products in anti-GBM patients during the GOOD-IDES-01 trial. (**A**) The ratio between C4d and C4 representing the CP and LP with rapid decreased activity after removal of autoantibodies by imlifidase, (**B**) the ratio between C3bBbP and C3 representing the AP, which remained on a similar elevated level throughout the trial and (**C**) the concentration of sTCC representing the terminal complement pathway in which activity decreased more slowly and remained elevated at the end of the trial. Significant change over time was tested using Friedman’s test with Dunns correction for multiple testing, *P < .05. As Friedman’s test requires complete data, one patient who died before the end of the trial was excluded, and single missing values have been interpolated linearly. Boxes represent quartiles, line represents median value and whiskers represent range.

As levels of complement proteins are affected by a wide number of factors, total C3 and C4 are not sensitive markers of complement activation. Instead, for activation of the CP we utilized the appearance of a neoepitope in C4d. However, since PLEX was used before and during the trial, we focused on the C4d/C4 ratio. The C4d/C4 ratio was higher before treatment (0.004, 0.000–0.058) compared with all following time points, reaching the .05 significance level in comparison with Day 22 (0.001, 0.000–0.004 *P *= .02) and Day 180 (0.000, 0.000–0.010, *P *= .003) (Fig. 1A). However, there was no linear relation (r^2^ < 0.1) between the C4d/C4 ratio and the levels of anti-GBM antibodies, either before treatment or at time points when rebound occurred (Days 7–29) (Supplementary data, Fig. S2A).

**Figure 2:**
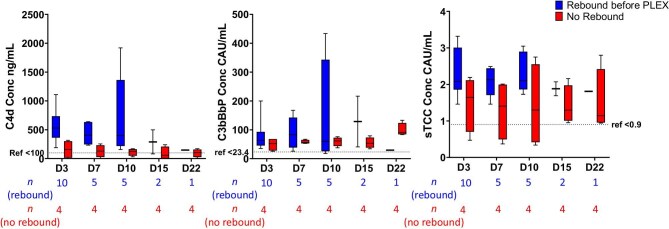
Comparison of complement activation products in anti-GBM patients with or without rebound of anti-GBM autoantibodies after removal by imlifidase. Concentration of C4d tended to be higher in patients with rebound (blue boxes) (**A**), C3bBbP concentration did not differ between patients with or without rebound (red boxes) (**B**), while a tendency to higher sTCC concentration was observed for patients with rebound (**C**). The number of patients, indicated below the X-axis for each group, in the rebound group (blue boxes) decreases over time because PLEX was restarted. Here, we utilized the absolute C4d and C3bBbP levels as values had not been distorted by PLEX. Mann–Whitney U-test with Hôlm Sídak correction for multiple comparisons did not reveal any significant differences between the groups at each timepoint. Boxes represent quartiles, line represents median value and whiskers represent range.

As rebound occurred at different time points, we also analyzed the last sample taken before restarting PLEX because of rebound. Here, we utilized the total C4d levels as values had not been distorted by PLEX. We observed a tendency to higher C4d levels in patients with rebound before PLEX was restarted (531 ng/mL vs 157 ng/mL, Day 3, *P *= 0.05) (Fig. 2A).

#### C3bBbP

For activation of the AP, we analyzed the appearance of the complex C3bBbP, and our primary analysis was the ratio between C3bBbP/C3. C3bBbP/C3 ratio remained unchanged throughout the trial (Fig. 1B), and there was no linear relation to levels of anti-GBM autoantibodies (Supplementary data, Fig. S2B). We could not see a tendency to higher C3bBbP levels in patients with rebound before PLEX was restarted (Fig. 2B).

#### sTCC and C5a

sTCC was elevated in comparison to the reference level (<0.9 CAU/mL) throughout the trial but tended to decrease over time and was significantly decreased from Day 22 (1.60 CAU/mL, 0.58–2.90, *P *= .01) and during the rest of the study as compared with before treatment (2.45 CAU/mL, 1.25–6.46) (Fig. 1C). There was no linear relation between sTCC and levels of anti-GBM autoantibodies (Supplementary data, Fig. S2C). We observed a tendency to higher sTCC levels on Days 3–10 in patients with rebound before PLEX was restarted (Fig. 2C). C5a levels varied between 4.6 and 48.6 ng/mL without any consistent pattern over time or in relationship to clinical parameters (Supplementary data, Fig. S3).

### Complement activation in relation to imlifidase

**Figure 3:**
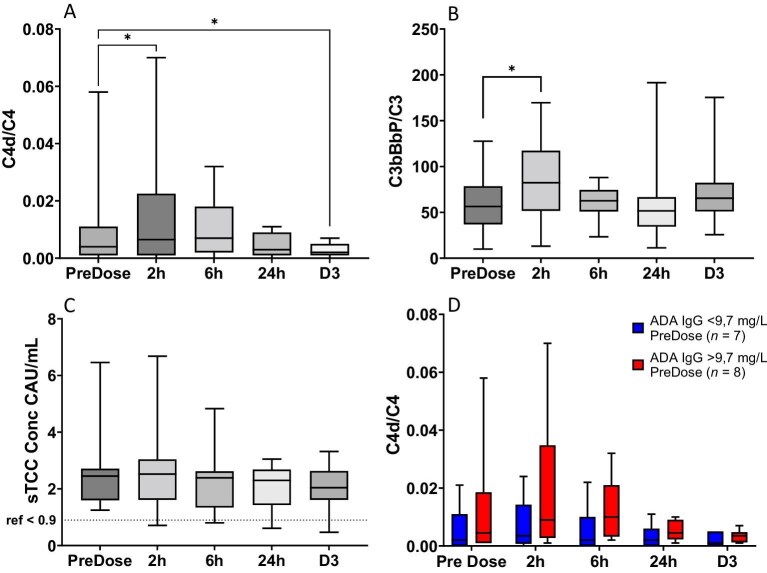
Complement activation products before and immediately following imlifidase treatment in anti-GBM patients in the GOOD-IDES-01 trial. There was a transient increase in the C4d/C4 ratio (**A**) and the C3bBbP/C3 ratio (**B**) but not in the concentration of sTCC (**C**) immediately following administration of imlifidase. The increase was more pronounced in patients with above median pre-existing ADA (red boxes) (**D**). In (A) to (C) Wilcoxon’s signed rank test was used to identify significant differences between timepoints, *P < .05. In (D) Mann–Whitney U-test with Hôlm Sídak correction for multiple comparisons did not reveal any significant differences between the two groups, nor did a mixed model analysis with Hôlm Sídak correction for multiple comparisons reveal any significant differences within each group over time. Boxes represent quartiles, line represents median value and whiskers represent range.

There was a significant increase in C4d/C4 (0.004 vs 0.007, *P *= .02) and C3bBbP/C3 (56.4 CAU/mg vs 82.3 CAU/mg, *P *= .03) but not STCC, 2 h after imlifidase administration. On Day 3, C4d/C4 was significantly lower than before treatment (0.002 vs 0.004, *P *= .03), indicating a transient early activation of the CP (Fig. 3A–C).

Low levels (9.7 mg/L, 3.5–16.6 mg/L) of ADA towards imlifidase were present before treatment with imlifidase in all patients, as previously reported [10]. ADA concentrations peaked on Day 15 (508 mg/L, 67–2822 mg/L) and remained on a similar level Day 50, but 3 months after treatment levels decreased (323 mg/L, 20–898 mg/L) (Supplementary data, Fig. S4).

The early activation of the CP tended to be more pronounced in patients with above median pre-existing ADA (increase of 0.004 vs 0.002 in the first 2 h, *P *= 0.2 using Mann–Whitney U-test and *P *= .1 using a mixed effects model) (Fig. 3D), but this was not the case for the AP (data not shown).

The pharmacokinetics of imlifidase differed substantially between patients, which is in line with previously published data [18]. Seven days after treatment, imlifidase was still measurable in seven patients (47%) in this study. At that time, ADA started to become measurable again. However, patients with remaining imlifidase concentration in the circulation at Day 7 did not exhibit higher levels of any of the three complement activation products, nor did they display an increased generation of ADA during the following 3 weeks (Supplementary data, Fig. S5).

Imlifidase concentration and ADA levels did not differ between patients with and without rebound of anti-GBM antibodies (data not shown).

### Complement activation in patients double positive for ANCA

We next compared complement activation in the six patients who were double positive for ANCA (DPP-ANCA), four of whom were myeloperoxidase (MPO)-ANCA positive and two proteinase 3 (PR3)-ANCA positive (Table 1). Higher levels of sTCC were observed in DPP-ANCA patients during the first 3 months (2.7 CAU/mL vs 1.7 CAU/mL Day 10, *P *= .005), but there were no differences in C4d/C4 or C3bBbP (Supplementary data, Fig. S5). ANCA returned after cleavage with imlifidase in three (50%) DPP-ANCA patients, but we did not observe differences in levels of complement activation products between DPP-ANCA with or without rebound of ANCA (data not shown).

**Table 1:** Characteristics of patients in the GOOD-IDES-01 trial.

	*n *= 15
Age, years, median (min, max)	61 (19, 77)
Gender	
Male, *n* (%)	9 (60)
Female, *n* (%)	6 (40)
Dialysis at inclusion	
No, *n* (%)	5 (33.3)
Yes, *n* (%)	10 (66.7)
Dialysis at 6 months	
No, *n* (%)	10 (66.7)
eGFR, mL/min/1.73 m, median (min, max)	26.4 (16.3, 59.1)
Yes, *n* (%)	4 (26.7)
Dead, *n* (%)	1 (6.7)
ANCA positivity	
No, *n* (%)	9 (60)
MPO, *n* (%)	4 (26.7)
PR3, *n* (%)	2 (13.3)
No. of PLEX sessions after inclusion, *n*, median (min, max)	3 (0, 18)

eGFR, estimated glomerular filtration rate.

### Complement activation in patients with anti-GBM autoantibodies of subclass IgG4

We found in a previous study [19] that in three patients, IgG4 was the dominating subclass of the anti-GBM autoantibodies. The level of complement activation products did not differ in these patients compared with the other 12 patients (data not shown).

### cfDNA

Finally, we analyzed the amount of cfDNA trying to establish a link between cell death and complement activation. During the first part of the trial mtDNA was volatile, with a low median at 6 h and a high median at Day 3. At 3 and 6 months both mtDNA and nDNA tended to be lower compared with before imlifidase treatment (Supplementary data, Fig. S6). The ratio of mtDNA/nDNA in DPP-ANCA patients was numerically lower but not significantly different (57 vs 190, Day 93, *P *= .2) (Supplementary data, Fig. S7). We did not observe differences in levels of cfDNA between patients with or without need for dialysis, either before treatment or at the end of the study (data not shown).

## DISCUSSION

In this study, we have explored complement activation in anti-GBM disease before and after treatment with imlifidase using ELISAs detecting complement fragments and complexes. We found that the C4d/C4 ratio, likely mostly representing the CP, decreased rapidly following autoantibody degradation by imlifidase. However, C3bBbP/C3, representing the AP, remained on a similar elevated level throughout the trial, while sTCC, representing terminal pathway stemming from any or all three pathways, decreased more slowly than C4d/C4 and remained above the reference level throughout the trial. This indicates that the CP activated by autoantibody binding to the GBM is of importance but that the AP and terminal pathway are also active in anti-GBM disease.

The importance of complement in anti-GBM pathogenesis has been proposed from induction of experimental autoimmune glomerulonephritis (EAG) in polymorphonuclear depleted animals [3]. C3 knockout mice and knockouts of the immunoglobulin receptor Fcγ developed less severe EAG, while this was not the case for C4 knockouts [9]. The fact that C4 knockouts are less protected from disease development supports the importance of the AP in disease development, though the relative importance of the complement pathways differs between species.

C4d can result from both the CP and LP, however the LP is likely to be less important in anti-GBM disease [9, 24]. Nonetheless, a biopsy study of anti-GBM disease patients showed that MBL deposition parallelled that of IgG and C3 [7] and IgG4 can activate complement by binding to MBL [6].

The C3BbBb fragment is a relatively short-lived complex in the bloodstream. However, measurement of C3bBbP provides unique insight into the central amplification step of the alternative pathway, which is not accessible via assays of only downstream fragments. The assay has previously been used in clinical samples, and in some cases the alternative pathway activation occurred in the absence of pronounced terminal pathway activation indicated by sTCC [25].

The importance of activation of the whole complement pathway, including sTCC, has been shown previously. sTCC is required for the full expression of EAG [26] and induction of glomerular endothelial apoptosis [27], both demonstrated using C6 knockout models. A positive correlation between sTCC and crescent formation has been found in anti-GBM patients [28]. Furthermore, experiments *in vitro* and *ex vivo* showed that complement activation on endothelial cell surfaces promotes neutrophil adhesion, which was significantly reduced by blocking sTCC formation [29]. Generation of sTCC is always accompanied by generation of C5a, but we did not see any correlation when analyzing this fragment, most probably due to rapid turnover and receptor binding of C5a.

Despite 6 months of observation, complement activation remained elevated throughout the trial. Perhaps this is partly explained by the observation that the autologous phase of EAG is negatively associated with complement [30], indicating an important role for complement in healing. Indeed, complement activation is crucial in both controlled apoptosis and clearance of debris, both important phenomena in the recovery phase [5]. Furthermore, complement, and the AP in particular, is active in the development of tubulointerstitial fibrosis associated with chronic kidney disease [31–33]. Although anti-GBM disease mainly affects the glomeruli in the acute phase, the glomerular damage leads to secondary tubulointerstitial changes, and it is possible that local activation of AP is the cause of the sTCC increase we see in this study. Using similar methodology in AAV we found in a recent study that sTCC was elevated during stable remission mainly in patients with renal impairment [34].

A possible explanation for continued AP activation and sTCC generation during convalescence is activation by cellular debris generated by tissue remodeling or processes such as NET-osis. To explore this hypothesis, we measured circulating cfDNA. Both sTCC and cfDNA decreased towards the end of the study, but we cannot draw any firm conclusions from these measurements, due to the small sample size and the short half-life of cfDNA making levels volatile [35].

We could not identify a linear relationship between any of the complement activation products and the level of anti-GBM antibodies. We did, however, observe that patients with rebound of anti-GBM tended to have higher levels of C4d and sTCC before rebound, indicating that there is in fact a relationship between the presence of anti-GBM autoantibodies and complement activation.

The influence of PLEX on complement components is not fully understood [6], but we reasoned that products of complement activation and their predecessors are likely to be removed to an equal degree by PLEX. We, therefore, opted to use ratios of the activation product and its predecessor to correct for any influence of PLEX. However, this was not possible for sTCC which consists of many components.

Immediately following imlifidase administration, a transient increase in activation of the CP was observed. This is probably due to preexisting ADA. ADA production increased after imlifidase but was not related to drug half-life, as production was not higher in patients with remaining imlifidase concentration in the circulation when ADA production resumed compared with patients with no remaining imlifidase. The first days after the initial imlifidase treatment, it could be feasible to give an additional dose of imlifidase, for example to patients with increased risk of rebound of autoantibodies, which we have previously shown is associated with a worse renal outcome [19]. The duration of this safe period cannot be established by the present study.

Our study has several limitations. Most importantly, the sample size is small and thus it is not possible to draw any firm conclusions. The risk of both type 1 and type 2 errors is considerable, especially as we perform multiple comparisons. Our results can only be viewed as hypothesis generating. Furthermore, complement analyses are in general sensitive to sample handling and storage, and despite our best efforts, we cannot exclude differences in sample handling between the local sites and the central laboratory. Our study also has strengths, most importantly that no other study has prospectively gathered clinical data and blood samples in anti-GBM disease before, allowing us to follow disease progression and healing process. Another strength when analyzing complement is using products of complement activation as they more specifically show the activity of the different pathways with fewer other factors affecting them.

In conclusion, we observed a rapid decrease of activation through the CP following autoantibody degradation by imlifidase, but a slower decrease in total complement activation which remained elevated throughout the trial. This indicates that therapies targeting complement could be of value in anti-GBM disease to improve renal outcome. We also detected a tendency to more activation of the CP before rebound of anti-GBM autoantibodies and a tendency to more total complement activation in DPP-ANCA. Immediately following imlifidase administration, we observed a transient increase in activation of the CP most probably due to preexisting ADA. This has could have importance when considering redosing of imlifidase.

## Supplementary Material

sfaf393_Supplemental_Files

## Data Availability

The data underlying this article will be shared upon reasonable request to the corresponding author.
