# Supplementary material for: Complement activation in anti-glomerular basement membrane disease before and after treatment with imlifidase
Source: Clin Kidney J. 2025 Dec 16;19(1):sfaf393. doi: 10.1093/ckj/sfaf393 (PMC12789867; doi:10.1093/ckj/sfaf393)
Supplement: sfaf393_Supplemental_Files [file sfaf393_supplemental_files.zip › 609 Supplementary_Methods_revised.docx]

# Supplementary Methods

## Measurement of antibodies and imlifidase concentration

Serum samples were analyzed for anti-GBM, ANCA and ANA antibody levels at the Department of Clinical Immunology and Transfusion Medicine, Region Östergötland, Linköping, Sweden using Thermo Fisher EliA. Anti-drug antibodies (ADA) directed against imlifidase was measured using ImmunoCAP (Thermo Fisher Scientific), as previously described (17). Serum concentration of imlifidase was determined using an electrochemiluminescence immunoassay, and the results were evaluated using WinNonlinProfessional (Pharsight Corporation, St Louis, MO) (18).

## Measurement of cell-free DNA

The amount of cell free mitochondrial and nuclear DNA (mtDNA and nDNA) was measured using droplet digital polymerase chain reaction (ddPCR), where the sample is fractionated into 20 000 droplets by water-oil emulsion and subsequently a PCR amplification of the template molecules occurs within each droplet. Droplets were generated using a BioRad (Hercules, CA, USA) QX200 droplet generator by adding sample, primers for mtDNA (BioRad, Cat #10031253) and nDNA (BioRad, Cat #10031244), restriction enzyme Hind III (New England Biolabs, Ipswich, MA, USA, Cat# R0104S) mixed with NE buffer r2.1 (New England Biolabs B6002S) and Supermix for probes (BioRad, L002699C) as well as droplet oil (BioRad, Cat# 1863005) into separate rows of a DG8^TM^ cartridge (BioRad, Cat# 1864008). Droplets were then transferred to a 96-well PCR plate (BioRad Cat# 12001925) which was sealed using PCR thermal foil (BioRad Cat# 1814040) a BioRad px1 plate sealer and placed in a thermal cycler with the following steps: activation at 95°C for 10 minutes, denaturation at 94°C for 30 seconds, 40 1-minute cycles of annealing/extension at 60°C, held at 98°C for 10 minutes and then cooled to 4°C. The plate was then kept at 4°C over night before readout using droplet reader oil (BioRad Cat# 1863004) and a BioRad QX200 droplet reader. A reference range for mtDNA and nDNA values has not been determined.

## Statistical analysis

Descriptive statistics were calculated in Microsoft Excel and GraphPad Prism version 9.0 (GraphPad Software, San Diego, CA, USA). Plots were created using GraphPad Prism version 9.0. Statistical analysis of changes over time were performed with Friedman’s test, Dunns correction for multiple comparisons, after linear interpolation of single missing values and exclusion of one patient who died before the end of the trial. Linear regression was used to investigate the relationship between anti-GBM concentration and the complement activation products. Differences between groups were analyzed using Mann-Whitney U-test with Holm-Sídak correction for multiple testing and Wilcoxon’s test was used to assess differences between timepoints. Additionally, differences between groups over time was evaluated with a mixed effects model with a group x time interaction using the nparLD package in RStudio version 2024.12.1 (Posit Software, Boston, MA, USA).
