# Supplementary material for: Complement activation in anti-glomerular basement membrane disease before and after treatment with imlifidase
Source: Clin Kidney J. 2025 Dec 16;19(1):sfaf393. doi: 10.1093/ckj/sfaf393 (PMC12789867; doi:10.1093/ckj/sfaf393)
Supplement: sfaf393_Supplemental_Files [file sfaf393_supplemental_files.zip › Supplementary Figures - revised.pdf]

Supplementary Figures

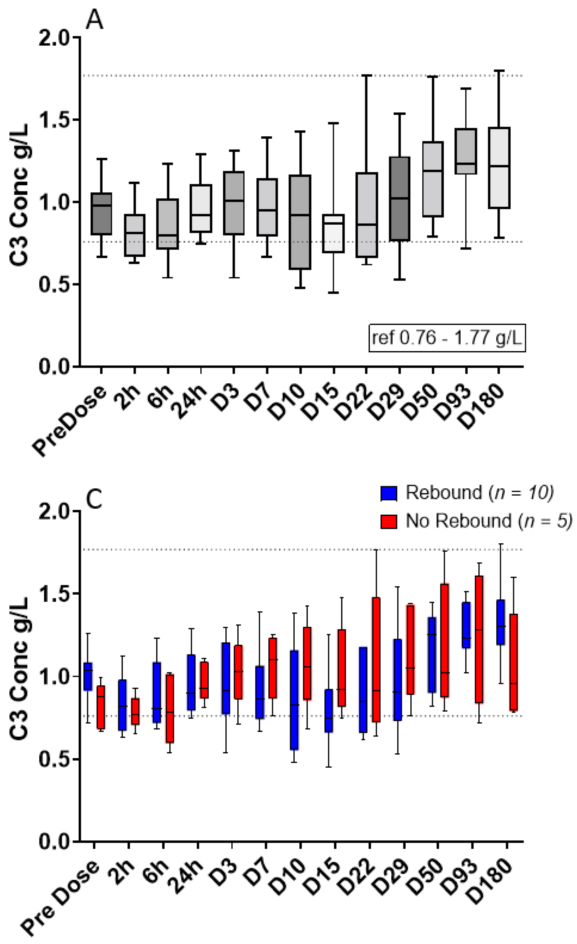

Supplemental figure 1 Plasma concentration of complement components in anti-GBM patients during the GOOD-IDES-01 trial. C3 was within normal range for most of the patients but was low in some patients before treatment and during the first four weeks after treatment (A), while C4 was within normal range for almost all patients throughout the trial (B). (C) shows a comparison of C3 concentrations between patients with (blue boxes) or without rebound (red boxes) of anti-GBM autoantibodies after removal by imlifidase (C). Boxes represent quartiles, line represents median value and whiskers represent range.

GBM = glomerular basement membrane

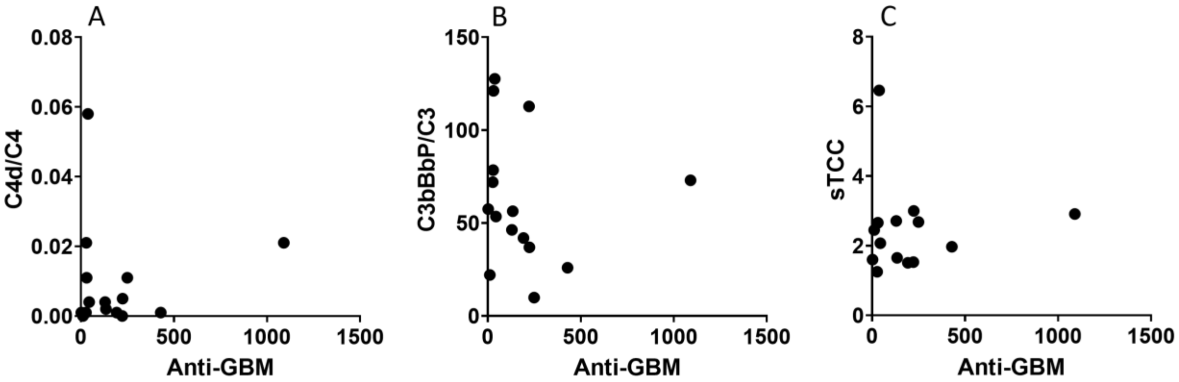

Supplemental figure 2 Relationship between concentration of complement activation products and anti-GBM autoantibody concentration. There was no clear linear relationship between the concentration of anti-GBM autoantibodies and the ratios of C4d/C4 (R^2 <0.01, Spearman's rho 0.2) (A), C3bBbP/C3 (R^2 =0.01, Spearman's rho -0.3) (B) or concentration of sTCC (R^2 <0.01, Spearman's rho 0.3) (C).

GBM = glomerular basement membrane;
sTCC = soluble terminal complement complex

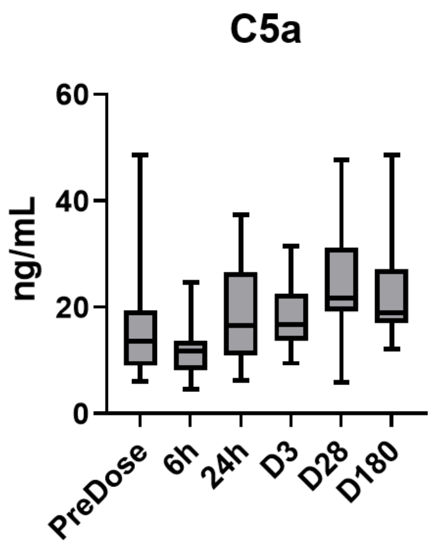

Supplemental Figure 3 C5a concentrations (ng/mL) in plasma from anti-GBM patients in the GOOD-IDES-01 trial measured by ELISA. C5a levels varied over time without a consistent pattern.

GBM = glomerular basement membrane;

ELISA = enzyme linked immunosorbent assay.

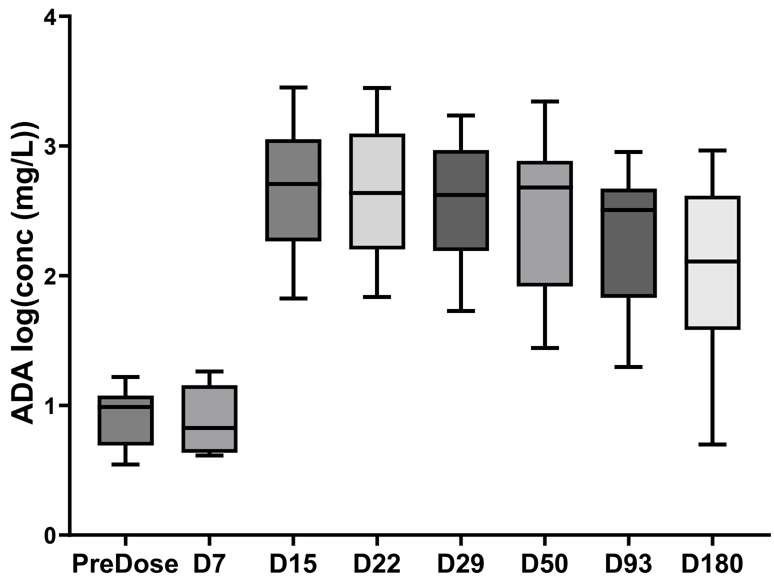

Supplemental figure ~~3~~ 4 Anti-drug antibody concentration in anti-GBM patients during the GOOD-IDES-01 trial, previously published data by Uhlin et al (10). All patients had some ADA before imlifidase treatment, and ADA levels increased after imlifidase treatment. Boxes represent quartiles, line represents median value and whiskers represent range.

ADA = anti-drug antibody;

GBM = glomerular basement membrane;

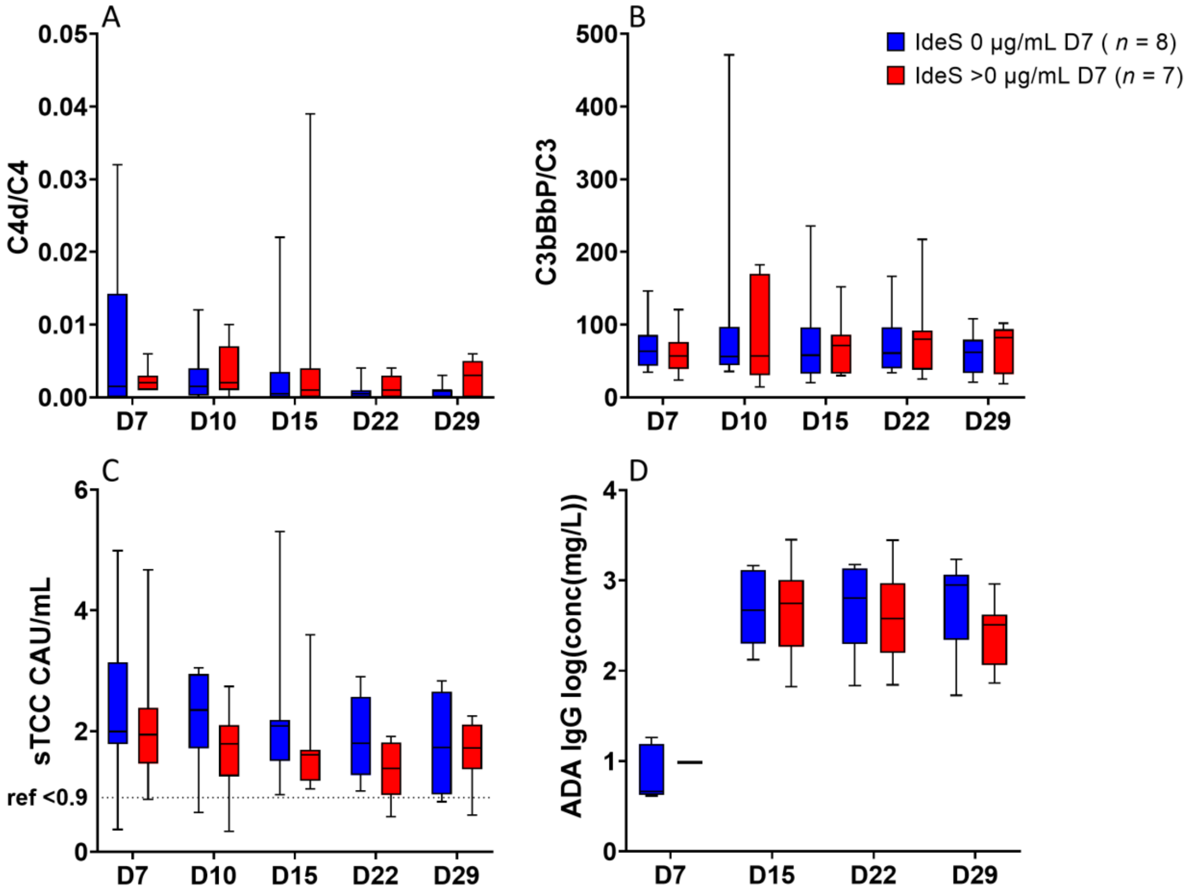

Supplemental figure 4 5 Complement activation and ADA production in anti-GBM patients with and without remaining imlifidase concentration seven days after administration. There was no difference in C4d/C4 ratio (A), C3bBbP/C3 ratio (B) or sTCC concentration (C) in patients with (red boxes) or without (blue boxes) imlifidase (based on the enzyme IdeS) left in the circulation 7 days after administration. There was no difference in the concentration of anti-drug antibodies towards imlifidase in patients with or without imlifidase in the circulation 7 days after administration when ADA production had started again (D). Mann-Whitney U-test with Hôlm Sídak correction for multiple comparisons did not reveal any significant differences between the two groups. Boxes represent quartiles, line represents median value and whiskers represent range.

ADA = anti-drug antibody;
GBM = glomerular basement membrane;
sTCC = soluble terminal complement complex

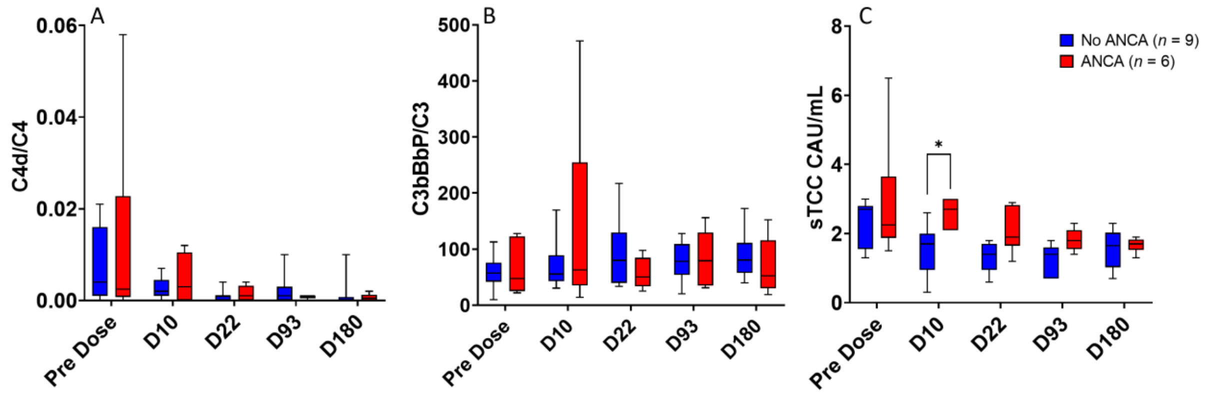

Supplemental figure ~~5~~ 6 Complement activation in anti-GBM patients with or without double positivity for ANCA. There was no clear difference between DPP-ANCA and single positive anti-GBM patients regarding the ratios of C4d/C4 (A) or C3bBbP/C3 (B) but there was a tendency to higher concentration of sTCC in DPP-ANCA (C). Mann-Whitney U-test with Hôlm Sídak correction for multiple comparisons, $p < 0.05$ indicated by *. Boxes represent quartiles, line represents median value and whiskers represent range.

GBM = glomerular basement membrane;
ANCA = anti-neutrophil cytoplasmic antibody;
DPP-ANCA = Double positive patient ANCA;
sTCC = soluble terminal complement complex

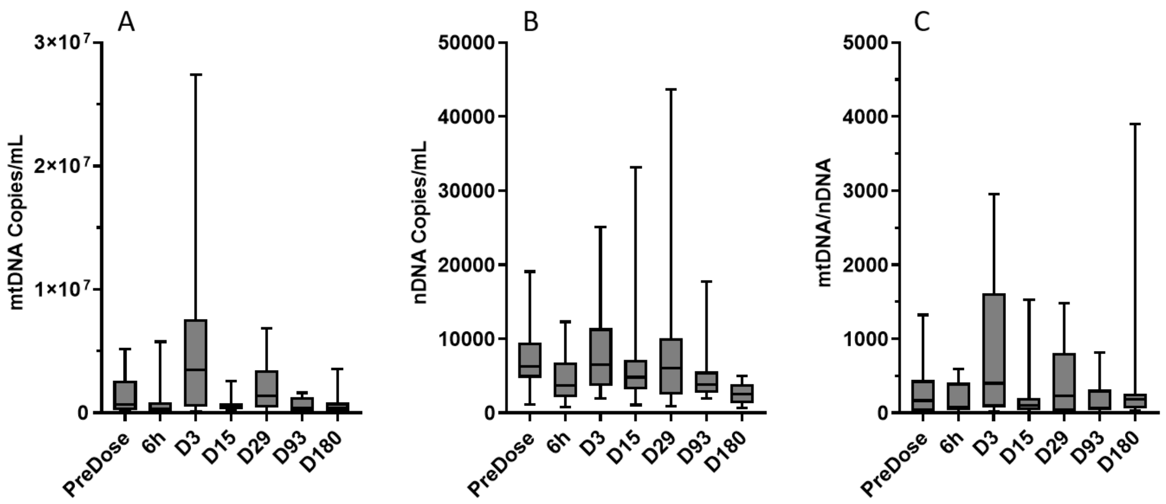

Supplemental figure ~~6~~ 7 Cell free DNA in anti-GBM patients during the GOOD-IDES-01 trial. The concentration of mtDNA (A) and nDNA (B) as well as the ratio of mtDNA/nDNA (C) was volatile during the first month of the trial but tended to be lower at three and six months compared to before treatment with imlifidase. Boxes represent quartiles, line represents median value and whiskers represent range.

DNA = deoxyribonucleic acid;
GBM = glomerular basement membrane;
mtDNA = mitochondrial DNA;
nDNA = nuclear DNA.

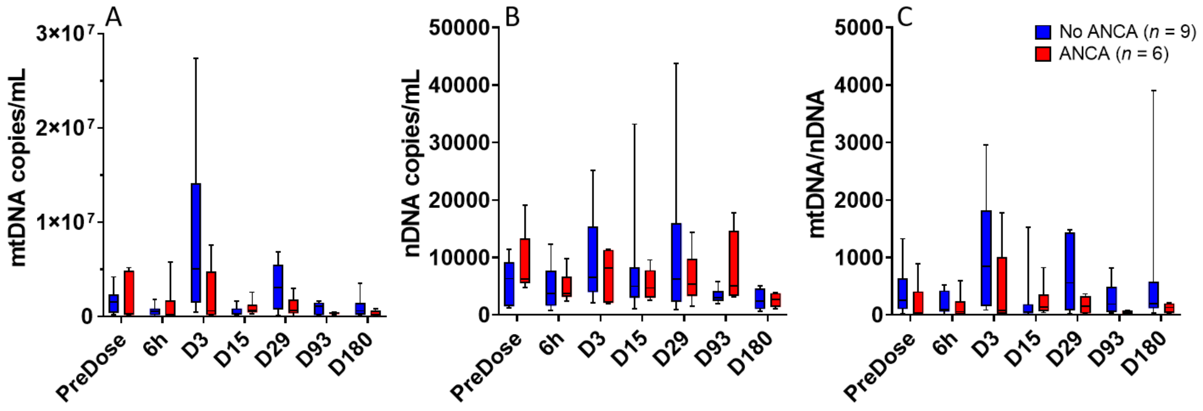

Supplemental Figure 8 Cell free DNA in anti-GBM patients in the GOOD-IDES-01 trial with or without double positivity for ANCA. The amount of mtDNA (A) and nDNA (B) was similar in DPP-ANCA and single-positive anti-GBM patients while the ratio of mtDNA/nDNA tended to be lower in patients with DPP-ANCA (C). Mann-Whitney U-test with Hôlm Sídak correction for multiple comparisons did not reveal any significant differences between the two groups. Boxes represent quartiles, line represents median value and whiskers represent range.

DNA = deoxyribonucleic acid;

GBM = glomerular basement membrane;

mtDNA = mitochondrial DNA;

nDNA = nuclear DNA;

ANCA = anti-neutrophil cytoplasmic antibody;

DPP-ANCA = Double positive patient ANCA
