# Supplementary figures and images for: Complement activation in anti-glomerular basement membrane disease before and after treatment with imlifidase
Source: Clin Kidney J. 2025 Dec 16;19(1):sfaf393. doi: 10.1093/ckj/sfaf393 (PMC12789867; doi:10.1093/ckj/sfaf393)

## Slide 1
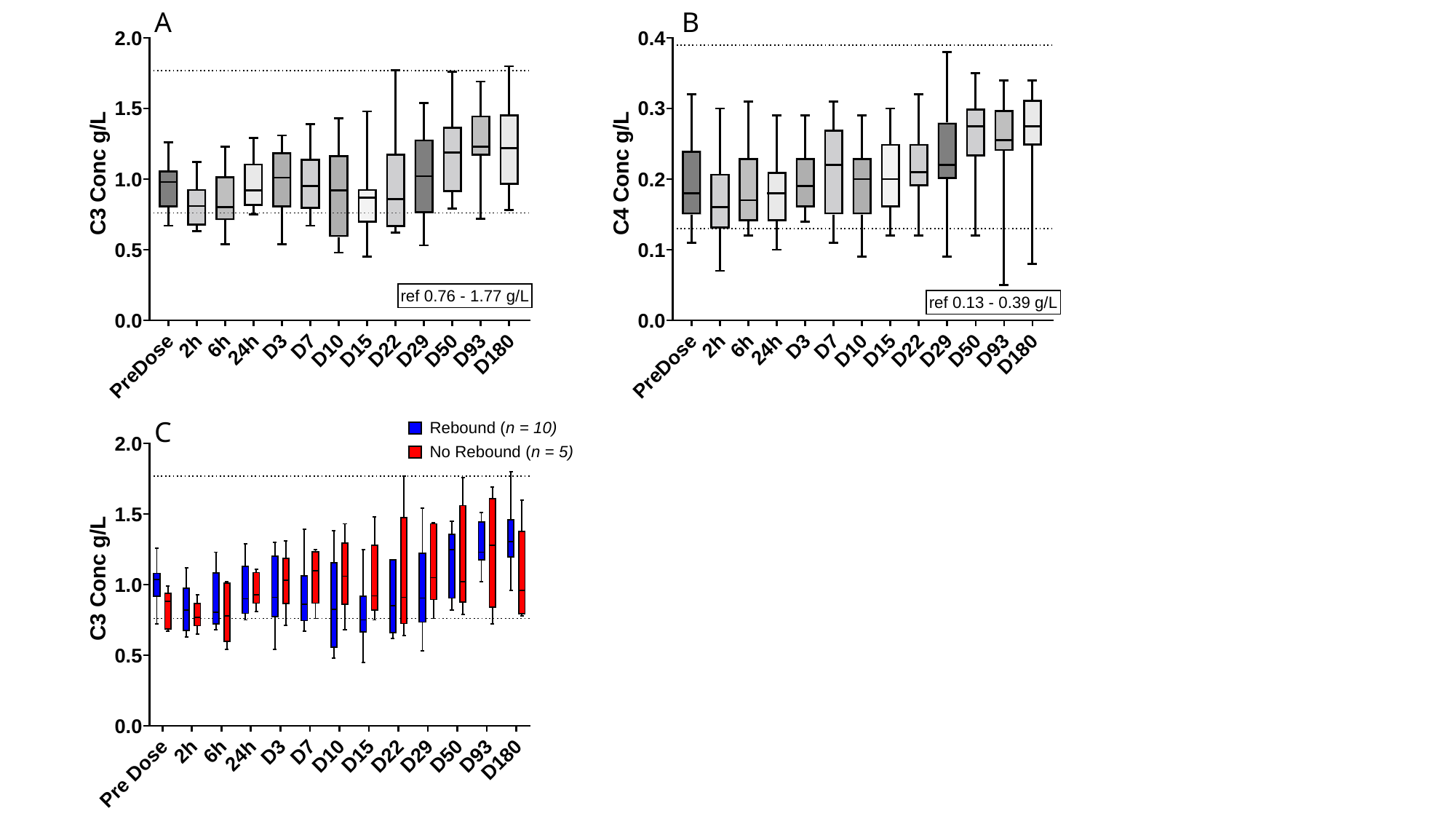

A
B
C

Supplement: sfaf393_Supplemental_Files [file sfaf393_supplemental_files.zip › Supplemental figure 1.pptx]

## Slide 1
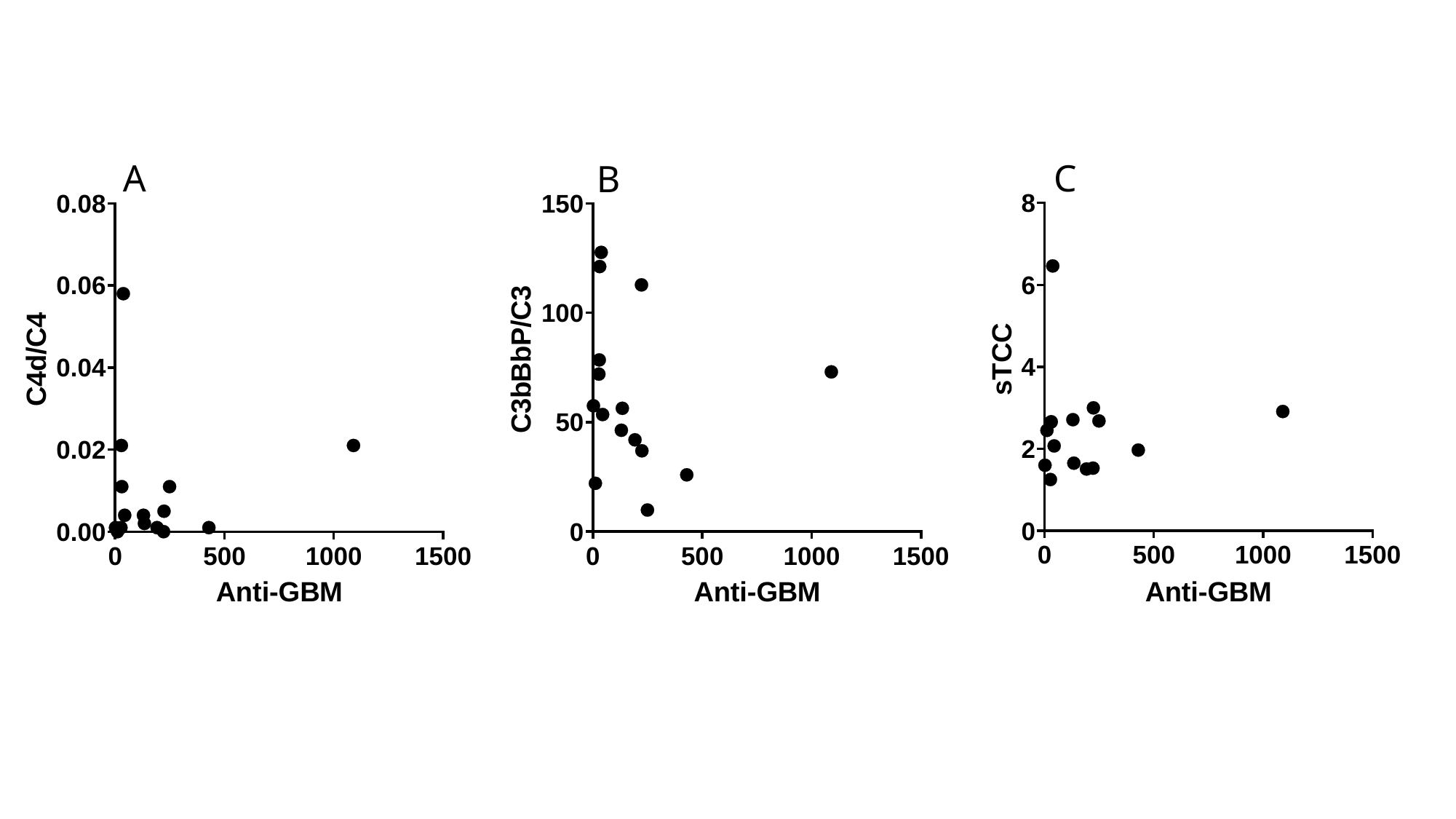

A
C
B

Supplement: sfaf393_Supplemental_Files [file sfaf393_supplemental_files.zip › Supplemental figure 2.pptx]

## Slide 1
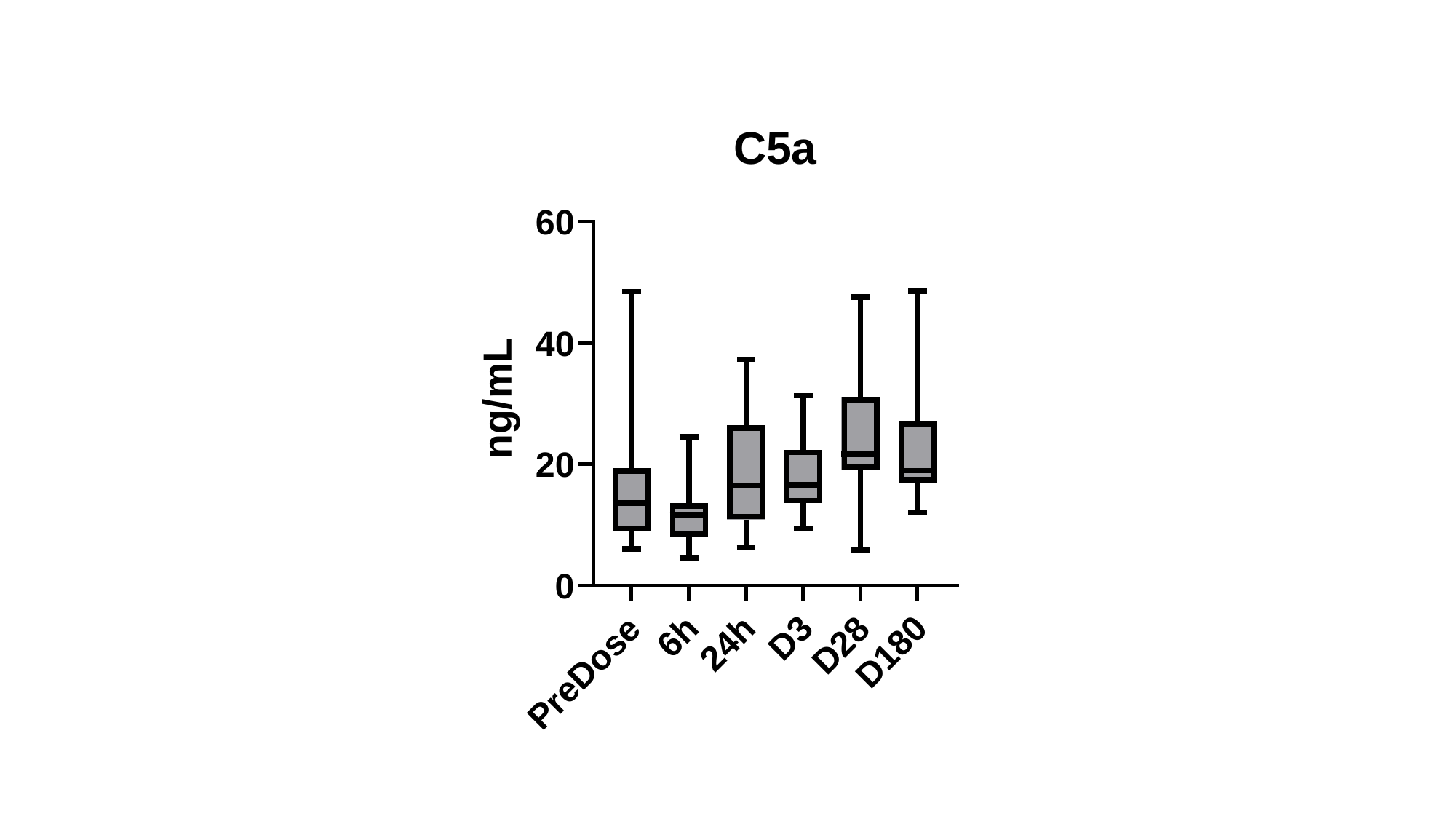

Supplement: sfaf393_Supplemental_Files [file sfaf393_supplemental_files.zip › Supplemental figure 3.pptx]

## Slide 1
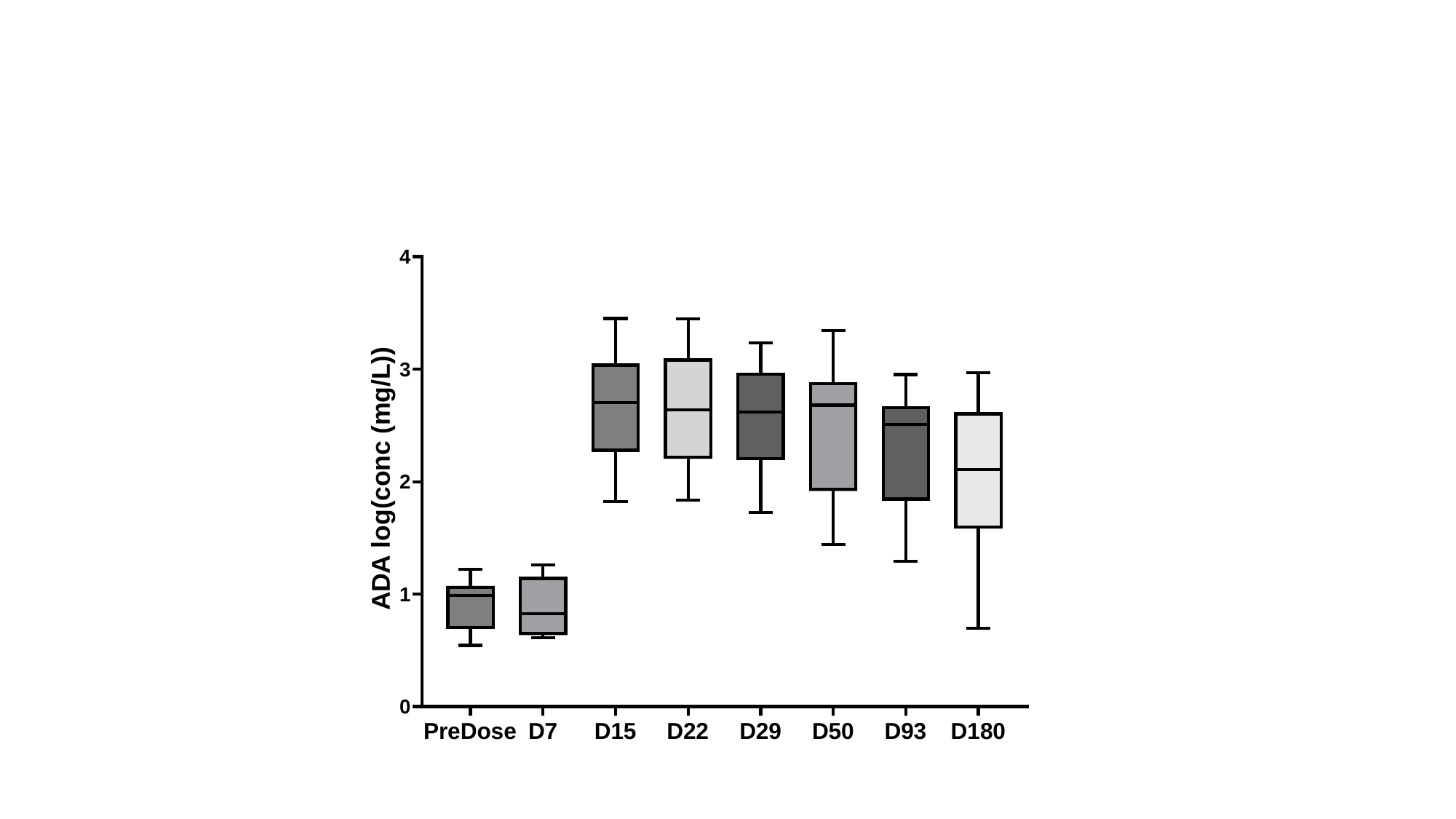

Supplement: sfaf393_Supplemental_Files [file sfaf393_supplemental_files.zip › Supplemental figure 4.pptx]

## Slide 1
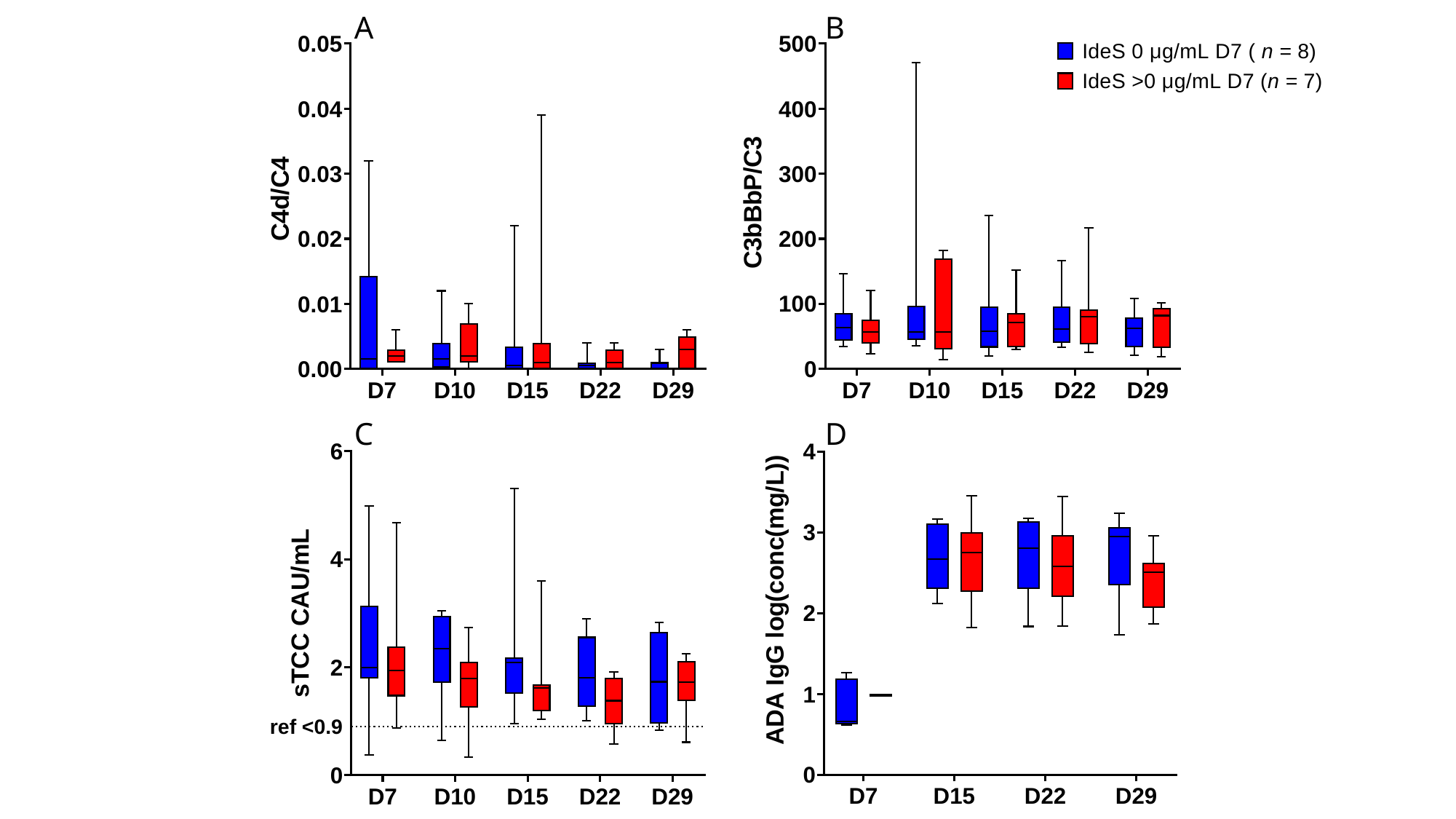

A
B
C
D

Supplement: sfaf393_Supplemental_Files [file sfaf393_supplemental_files.zip › Supplemental figure 5.pptx]

## Slide 1
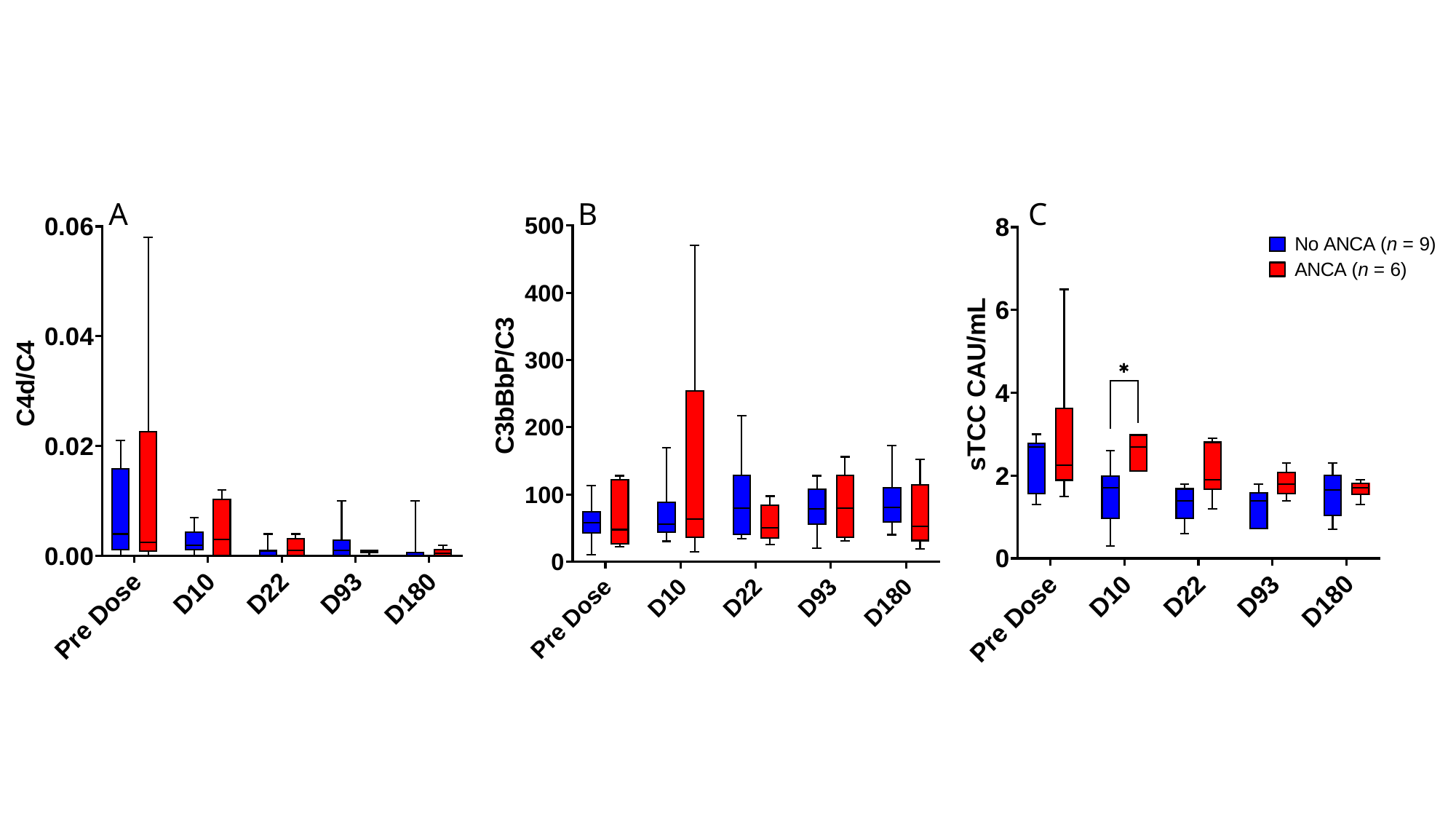

A
B
C

Supplement: sfaf393_Supplemental_Files [file sfaf393_supplemental_files.zip › Supplemental figure 6.pptx]

## Slide 1
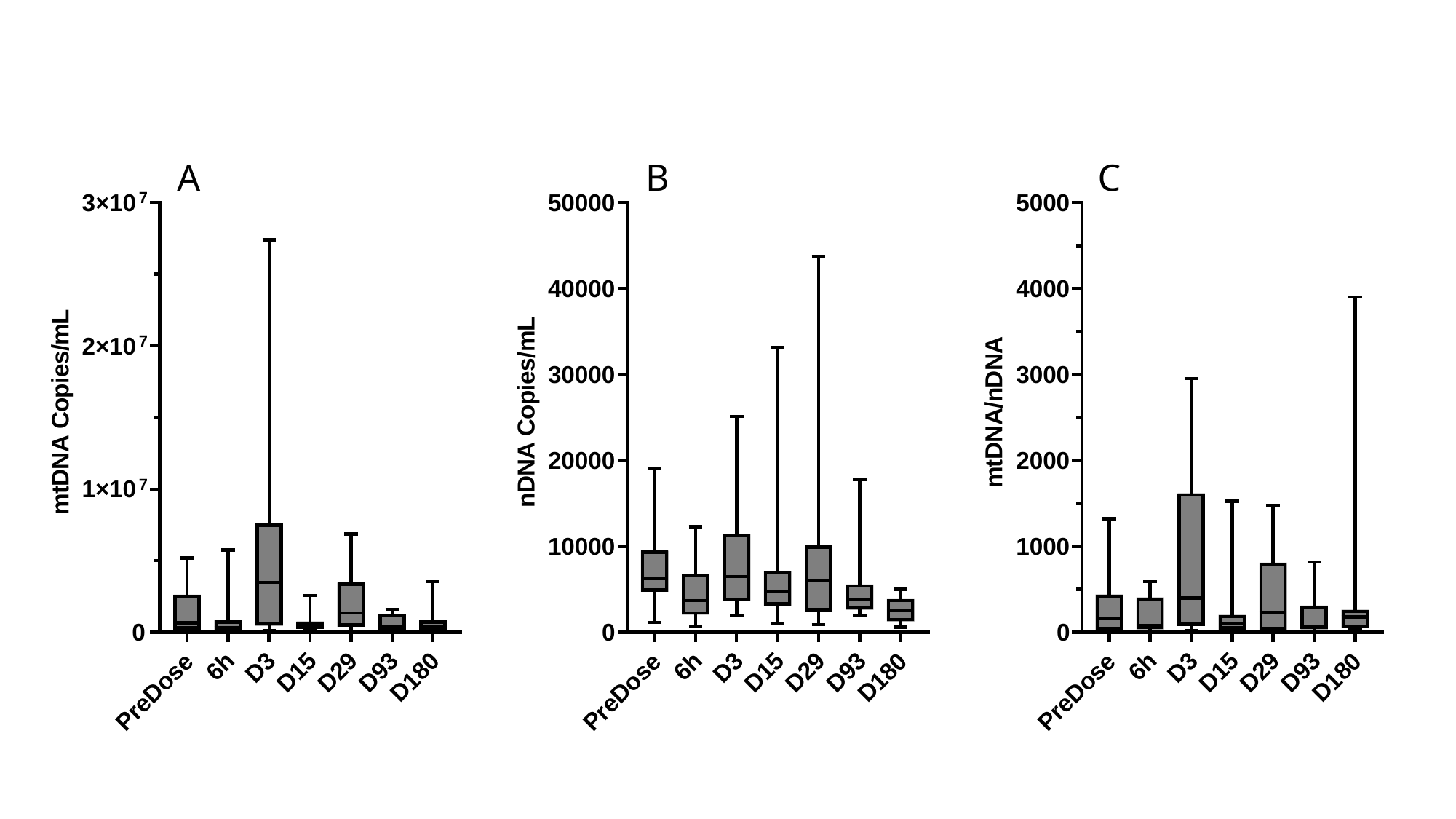

A
B
C

Supplement: sfaf393_Supplemental_Files [file sfaf393_supplemental_files.zip › Supplemental figure 7.pptx]

## Slide 1
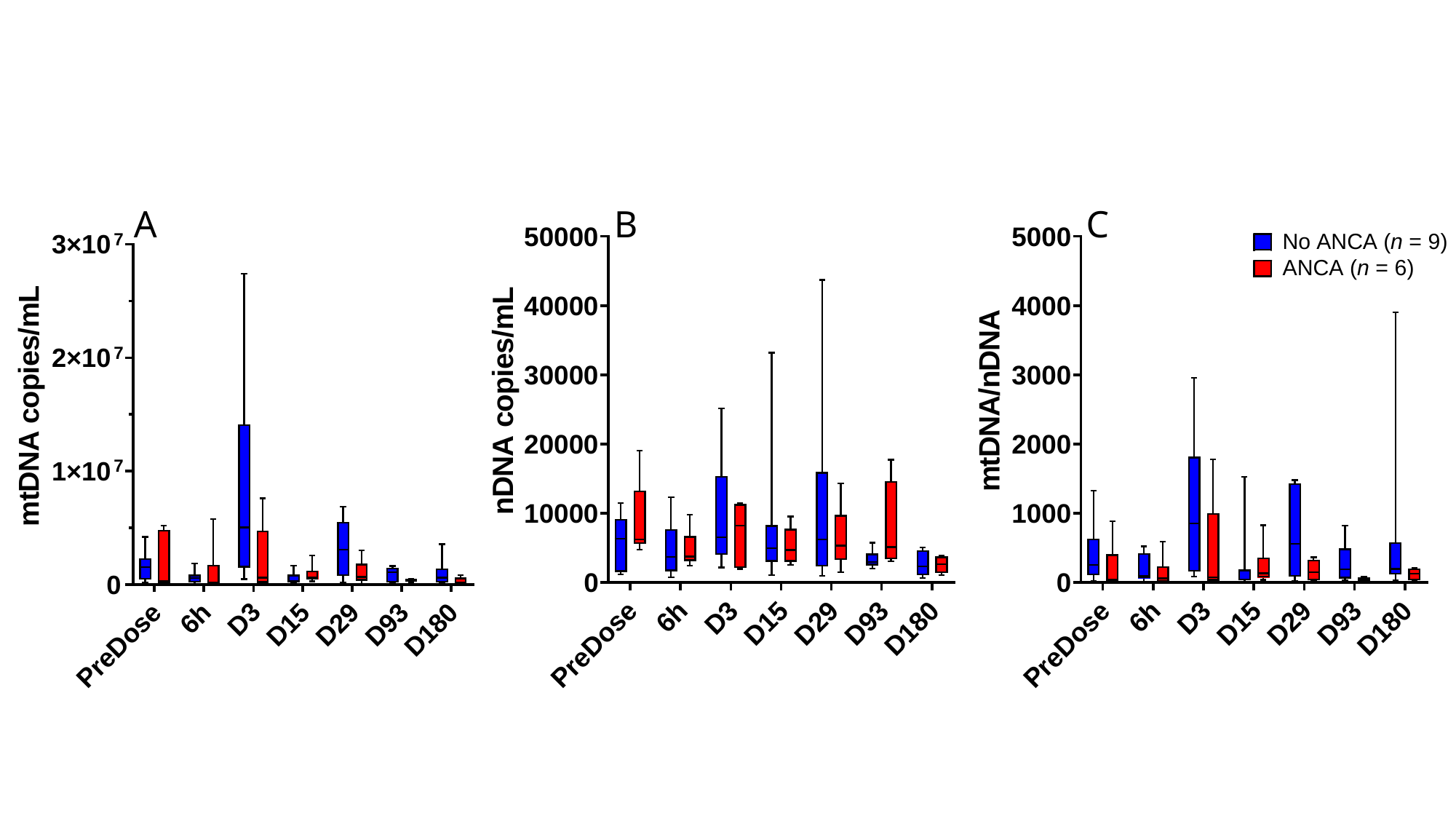

C
A
B

Supplement: sfaf393_Supplemental_Files [file sfaf393_supplemental_files.zip › Supplemental figure 8.pptx]
